# [Retracted] Effect of microRNA-27b on cisplatin chemotherapy sensitivity of oral squamous cell carcinoma via FZD7 signaling pathway

**DOI:** 10.3892/ol.2024.14408

**Published:** 2024-04-22

**Authors:** Bingyao Liu, Gang Cao, Zhen Dong, Ting Guo

Oncol Lett 18: 667–673, 2019; DOI: 10.3892/ol.2019.10347

Following the publication of this paper, it was drawn to the Editor's attention by a concerned reader that certain of the colony formation assay data shown in Fig. 2, the scratch-wound assay data in Fig. 3, the flow cytometric data in Fig. 5 and the Hoechst 33258-stained images in Fig. 6 were strikingly similar to data appearing in different form in articles written by different authors at different research institutes that had already been published. Owing to the fact that the contentious data in the above article had already been published prior to its submission to *Oncology Letters*, the Editor has decided that this paper should be retracted from the Journal. Independently of the reader's enquiry, the authors also requested that the paper be retracted. The Editor apologizes to the readership for any inconvenience caused.

